# Evaluating the Implementation of a Remote-Monitoring Program for Chronic Obstructive Pulmonary Disease: Qualitative Methods from a Service Design Perspective

**DOI:** 10.2196/18148

**Published:** 2020-10-09

**Authors:** Florence van Lieshout, Rebecca Yang, Vess Stamenova, Payal Agarwal, Daniel Cornejo Palma, Aman Sidhu, Katrina Engel, Adam Erwood, R Sacha Bhatia, Onil Bhattacharyya, James Shaw

**Affiliations:** 1 University of Amsterdam Amsterdam Netherlands; 2 Women's College Hospital Institute for Health System Solutions and Virtual Care Toronto, ON Canada; 3 Markham Stoufville Hospital Markham, ON Canada; 4 University of Toronto Joint Centre for Bioethics Toronto, ON Canada

**Keywords:** service design, digital health, innovation, implementation science, remote monitoring, telemedicine

## Abstract

**Background:**

Implementing digital health technologies is complex but can be facilitated by considering the features of the tool that is being implemented, the team that will use it, and the routines that will be affected.

**Objective:**

The goal of this study was to assess the implementation of a remote-monitoring initiative for patients with chronic obstructive pulmonary disease in Ontario, Canada using the Tool+Team+Routine framework and to refine this approach to conceptualize the adoption of technologies in health care.

**Methods:**

This study was a qualitative research project that took place alongside a randomized controlled trial comparing a technology-enabled self-monitoring program with a technology-enabled self- and remote-monitoring program in patients with chronic obstructive pulmonary disease and with standard care. This study included interviews with 5 remote-monitoring patients, 3 self-monitoring patients, 2 caregivers, 5 health care providers, and 3 hospital administrators. The interview questions were structured around the 3 main concepts of the Tool+Team+Routine framework.

**Results:**

Findings emphasized that (1) technologies can alter relationships between providers and patients, and that these relationships drove the development of a new service arising from the technology, in our case, and (2) technologies can create additional work that is not visible to management as a result of not being considered within the scope of the service.

**Conclusions:**

Literature on the implementation of digital health technologies has still not reconciled the importance of interpersonal relationships to conventional implementation strategies. By acknowledging the centrality of such relationships, implementation teams can better plan for the adaptations required in order to make new technologies work for patients and health care providers. Further work will need to address how specific individuals administering a remote-monitoring program work to build relationships, and how these relationships and other sources of activity might lead to technological scope creep—an unanticipated expanding scope of work activities in relation to the function of the tool.

## Introduction

The digital health market continues to expand, with projected growth from US $79 billion in 2015 to an estimated value of US $206 billion in 2020 [1]. This growth is driven, in part, by the fact that many patient-facing digital health technologies, such as remote monitoring, have shown potential for positive effects on patient health and health care provider performance, such as enhanced chronic disease management and enhanced access to care [2,3]. However, despite some demonstrated successes with digital health technologies, positive results remain sporadic and difficult to achieve [4].

The implementation of digital health technologies remains an extremely complex area of health system improvement, and remote monitoring has served as an important example of the challenges associated with encouraging the meaningful use of technologies in routine care [5,6]. The need for patients to feel comfortable with a given technology in addition to the health care provider team engaging in remote care delivery creates additional barriers to successful adoption [7,8]. Furthermore, many technologies are advertised to be “plug and play,” suggesting there should be no barriers to their immediate use when, in fact, much local adaptation is required for them to be incorporated into users’ everyday lives [9]. These observations illustrate the persistent and unresolved issues associated with determining both the mechanisms through which remote monitoring works for users and the optimal strategies to promote implementation.

Unsuccessful implementation of technologies such as remote monitoring is a result of several complex influences [10], including poor alignment with the main objective of the health care organization, poor training of staff, nonalignment of procurement models, or noninteroperability of the technology with existing systems [11,12]. Over the past 10 years especially, methods and frameworks have been developed to support better implementation of technological tools in health care settings [10,13,14]. Many of these approaches have been rooted specifically in theories of implementation science, including the Consolidated Framework for Implementation Research [15] and the Normalization Process Theory [16]. Theories of implementation science have been important in clarifying the conceptual dimensions requiring consideration in technology implementation initiatives. However, we suggest they have done less to clearly articulate the most important links between the adoption of a technology and the redesign of the service into which a technology is being implemented.

This basic point formed the motivation for previous work by our research team to present an approach to the adoption of digital health technologies that emphasizes the links between technological innovations and service innovations, informed by a service design perspective [17]. Service design is an approach to the improvement or establishment of services that takes the entire service as the primary focus, emphasizing the importance of user experience and the realities of service delivery for health care providers and other staff [17]. In our past work, we formulated the objective of service design: to “carefully plan and promote the coordinated action required to execute a high quality health care service [17].” In this way, service design considers a technology for its contributions to a service more generally by focusing less on the adoption of the technology itself and more on its contributions to the work required to deliver an excellent service overall.

The recently developed Tool+Team+Routine framework [17] outlines a service design approach to innovation adoption that includes (1) identifying the value propositions of a given tool, (2) considering the implications of the tool for team relationships, and (3) explicitly addressing the new routines required in the technology-informed reconfiguration of the service. Although this approach is based on past empirical research examining this alternative approach to technology adoption, the Tool+Team+Routine framework is a simple heuristic that has not yet been subject to critical examination [17].

The goals of this study were two-fold. First, to apply the Tool+Team+Routine framework to interpret the results of the implementation of a remote-monitoring initiative for patients with chronic obstructive pulmonary disease (COPD) in Ontario, Canada, in order to generate insights that inform potential improvements to the Tool+Team+Routine framework. Second, to identify insights pertaining to the influences that drive adoption of remote-monitoring technologies specifically.

## Methods

### Study Setting

This is a qualitative substudy of a randomized controlled trial [18]; both the randomized controlled trial and this qualitative study were conducted at a community-based hospital in a large city in Ontario, Canada, within a respiratory center that treats patients with COPD. The randomized controlled trial investigated the effectiveness of implementing a technology-enabled self-monitoring program or a technology enabled self- and remote-monitoring program in a COPD patient population compared to standard care. Both the self-monitoring and the remote-monitoring groups used the Cloud DX Connected Health Kit as the monitoring tool [19]. This tool consisted of a tablet and multiple smaller devices to measure physiological vitals and to complete surveys on objective COPD symptoms. The tablet notified patients whenever their readings exceeded their personal thresholds.

All patients from both groups were also provided with a paper-based action plan, which they could follow whenever they received such notifications. The vitals and questionnaire answers from the remote-monitoring group were monitored by the project lead who is also a respiratory therapist. If it was thought to be necessary by the respiratory therapist, based on the vitals or the questionnaire answers, the patient was be contacted directly. In addition, the project lead called each remote-monitoring patient once a week.

The study was approved by Markham Stouffville Hospital and Women’s College Hospital research ethics boards in Ontario, Canada.

### Participants

We included 8 patients with COPD (5 from the remote-monitoring group and 3 from the self-monitoring group), 5 health care providers, and 3 hospital administrators (herein referred to as hospital managers). One remote-monitoring patient and 1 self-monitoring patient were interviewed together with their caregiver. Participants were purposefully sampled in order to capture a meaningful and varied range of views on the program from the parties most intensively involved in its implementation. The patients were recruited by a respiratory therapist at the host site and were selected from the randomized controlled trial’s group of participants. These patients’ contact details were passed onto the research team. The patients were contacted for participation by the principal author (FvL), either over the phone or through email. Prior to the interview, all participants provided informed consent to participate and were informed that they could decline or withdraw from the study at any time without any consequence to their health care. Written or verbal consent was obtained from all participants by the principal author (FvL) at the start of the interview. All patients had moderate (Global Initiative for Chronic Obstructive Lung Disease level 2) to very severe (Global Initiative for Chronic Obstructive Lung Disease level 4) COPD [20]. Patients’ demographics were obtained from the randomized controlled trial’s onboarding questionnaires

### The Framework

The framework used in this research study was the Tool+Team+Routine framework [17]. Unlike other implementation frameworks, this framework focuses explicitly on service design. Some key ideas that characterize service design in health care are

1. Focus on all the changes to services that are required when introducing a new digital health tool in a health care delivery process.

2. A technology is not immutable; it may need to be modified in order to be of most use to the health care delivery process.

3. There needs to be clear value to all stakeholders, so-called *value propositions* [21]. Ideally, the new service improves one or more factors of the health care quadruple aim—enhancing patient experience, improving population health, reducing costs, and improving the work life of health care providers [22].

**Figure 1:**
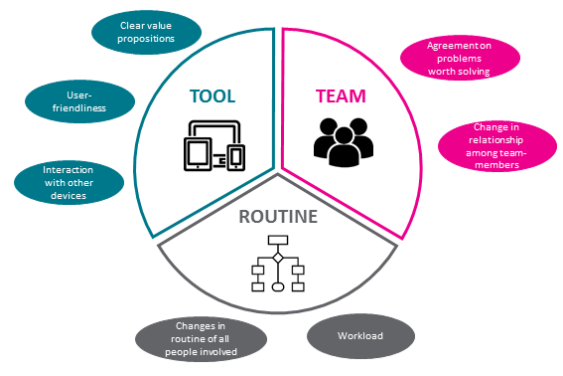
Visual representation of the main factors in the Tool+Team+Routine framework.

Implementing these principles of service design in health care, the Tool+Team+Routine framework is a simple, heuristic approach for introducing new digital health tools to a health care delivery process, or re-designing the delivery process overall and focuses on 3 main factors (Figure 1; Table 1).

**Table 1:** Tool+Team+Routine framework.

Factor	Description	Questions
Tool	Determines how useful and acceptable the technology is	Are the value propositions clear for all the stakeholders that will use the tool? Is the tool user-friendly? In what way does the tool interact with other devices?
Team	Investigates what changes will occur to the whole team and the way they interact with each other	Does the whole team agree on the problem to be solved by the technology? How does the introduction of the new system change the relationships among team-members, including changed relationships between patient and health care provider?
Routine	Considers in what way people’s routines change when the new system is introduced	What other changes will occur in the routines and day-to-day lives of all the people involved—intentionally or unintentionally—when the new system is introduced?

### Data Collection Methods

Participants were interviewed by the principal author (FvL). The interviews were semistructured and contained questions pertaining to the 3 main concepts of the framework. Each group of participants (patients, health care providers, and hospital managers) were interviewed using a different interview guide. Interviews lasted 30 to 60 minutes. All patient interviews were conducted over the phone. The interviews with the health care providers and hospital managers were conducted in person, except for 2 that were conducted by phone. All interviews were audiorecorded and transcribed verbatim. The transcripts were anonymized and deidentified.

### Data Analysis

The interview data were analyzed using thematic analysis methods [23,24]. The first 2 transcripts were analyzed and coded individually by the principal author and 2 other members of the research team. The codes were then compared and discussed which informed the development of a code book that was used to code 3 other transcripts. Thereafter, the code book was discussed again between the same 3 researchers, which resulted in the revised code book. This method was used to ensure the members of the research team contributing to the analysis agreed on the relevance and definitions of the codes being used. The remaining transcripts were analyzed and coded by the principal author using the revised code book. Any necessary changes to the coding of the first 5 transcripts that resulted from the revised code book were made as well, through regularly occurring analysis meetings between 4 authors on the study team.

## Results

### Overview

Table 2 provides an overview of the patients and caregivers who participated.

Table 3 provides an overview of the health care providers and hospital managers who participated. The tables provide the overall attitude toward the program (negative, neutral, mixed, or positive) as gleaned from participant interviews.

**Table 2:** Patients and caregivers who participated.

Participant	Study arm (remote or self-monitoring)	Gender	Age (years)	Interviewed with caregiver	Overall attitude toward program
P1	Remote monitoring	Male	73	No	Positive
P2	Remote monitoring	Female	89	No	Positive
P3	Self-monitoring	Female	69	No	Neutral
P4	Remote monitoring	Female	72	No	Neutral
P5	Remote monitoring	Male	65	No	Positive
P6	Self-monitoring	Female	75	No	Positive
P7	Remote monitoring	Male	73	Yes	Positive (patient); positive (caregiver)
P8	Self-monitoring	Female	81	Yes	Neutral (patient); positive (caregiver)

**Table 3:** Overview of the health care providers and hospital managers who participated.

Participant label	Job title	Level of interaction with Cloud DX^a^	Overall attitude toward program
HCP 1	Physician	3	Positive
HCP 2	Physician	3	Neutral
HCP 3	Allied health professional	5	Mixed
HCP 4	Allied health professional	5	Positive
HCP 5	Allied health professional	4	Positive
HM 1	Senior manager	2	Positive
HM 2	Manager	2	Positive
HM 3	Senior manager	1	Positive

^a^Levels—1 (very little): no interaction with the Cloud DX data on a daily basis and little to moderate understanding of the tool; 2 (little): no interaction with the Cloud DX data on a daily basis but moderate to high understanding of the tool; 3 (moderate): regular indirect interaction with the Cloud DX data but not involved in daily monitoring of patients; 4 (high): daily direct interaction with the Cloud DX data but not involved in daily monitoring of patients: 5 (very high): daily monitoring of patients with the Cloud DX tool and data.

In the sections below, qualitative descriptions of the results that emerged from the interviews are given. These results are structured around the 3 main themes of the framework: *Tool*, *Team*, and *Routine*. Verbatim quotations are labeled with the role of the participant and their study arm (for patient and caregiver participants) or their role and level of interaction with the Cloud DX tool (for health care provider and hospital manager participants).

### Attitude Toward the Project

Within all stakeholder groups, the attitude toward the project was mainly positive. Of the remote-monitoring patients, 4 out of 5 viewed the program as positive; the fifth had a neutral attitude toward the program. Of the self-monitoring patients, 1 out of 3 viewed the program as positive; the other 2 had neutral feelings about it. The 2 caregivers were both positive about the program. Within health care provider and hospital manager groups, attitudes toward the program were more nuanced. All 3 hospital managers showed positive attitudes toward the program. Of the health care providers, 3 out of 5 showed positive attitudes as well; the other 2 had mixed feelings about the program. These are elaborated.

### Tool: Value Propositions in a Technology-Push Project

Stakeholders’ perceptions of the value of the tool varied and were characterized by the fact that the implementation project was structured using a *technology-push* approach. In this case, a team of managers and providers from 2 different health care organizations established a project agreement with the vendor, securing funding to support an evaluation of the tool in a hospital environment. One manager explained expectations for the project:

I think [the technology] is allowing patients to catch their symptoms, uh sooner so they can start treatment before they get really sick. I think it’s keeping them away from the emergency department and it should be decreasing the number of admissions... So, decreasing admissions to hospital, overall, it’s gotta be improving their quality of life.

Senior manager, very little interaction

Only after the funding was in place and the agreement to complete the project was secured did the project team seek to engage the health care providers who would be using the tool in the newly established model of care. As a result, not all health care providers saw value in the tool, leading some to resist engaging with the technology in a meaningful way.

Resistance to engaging with the technology and its outputs was reported by some of the respirology physicians who were involved in the care of patients enrolled in the program. One physician stated:

I was very skeptical when the study was first started because uh, a lot of the times, these sort of home monitoring or self-monitoring or other programs kind of invent the technology first before asking a lot of important clinical questions of whether it’s actually gonna be of benefit or uh, examining why patients actually have exacerbations or the mechanisms and then it’s sort of just implemented into this study and then we see what happens, so, it’s uh, a bit of the uh, cart before the horse kind of scenario

Physician, moderate interaction

This quotation illustrates the general lack of engagement with the tool among some physicians primarily as a result of feeling that the tool does not solve a direct clinical need (although the lack of time physicians have for such initiatives is also clearly acknowledged). The primary example of this was that vitals data, such as blood oxygenation saturation, which are a primary data output of the tool for health care providers are known to be a poor predictor of exacerbations of COPD. Physicians were not satisfied with the effort to establish a meaningful use case for the tool after the decision to move forward with the project was made. This created a scenario where physicians had widely varying levels of engagement with the lead respiratory therapist who provided them with information about the outputs of the tool in order to inform their approach to care.

In this initiative, the physicians were not actually required to interact directly with the tool. Instead, a respiratory therapist was responsible for communicating with patients by phone regarding the tool and managing the inputs arising from patient data being collected at home. This respiratory therapist would communicate with physicians when remote-monitoring data from patients under their care suggested that some form of physician follow-up would be appropriate. Although the respiratory therapist had reservations about the workload implications of the tool, this person believed there would be a benefit for certain patients:

So the program I think it’s helping patients, ehm, better understand their condition, or at least some of the patients. Perhaps not all of them. And helping them to recognize when they’re getting sick so that they can get treatment, either sooner or more appropriately... I would say that some patients did, or were good self-recognizers [already] and those are patients who have come to the hospital one or two times before because they had experienced [an exacerbation]... However those who haven’t had many experiences with exacerbations, it helps them better recognize it.

Allied Health Professional, very high interaction

The patients and caregivers interviewed for the study reported some technology-related challenges that might be expected when adopting new digital tools for the first time. However, many participants reported generally positive sentiments about the tool and its adoption into their everyday lives. One patient, a woman in her 70s who lived alone, stated:

The tablet gave me, just that confidence because I could see that my blood pressure was in normal range though I kind of figured that. And then my oxygen levels, I didn’t know about my oxygen level and then, once I, I got to reading my oxygen levels, everything seems to be fine. So that, that was just, I don’t know, just a report back to myself saying “Hey, you’re not too bad at all”

Patient, remote monitoring

Another patient described an instance where her decision to seek out emergency services was directly informed by the tool. Although this example is not directly related to a patient’s experience of COPD, people living with COPD often experience co-occurring conditions such as atrial fibrillation.

[Respondent:] ... my heart was beating too fast and because I had the, eh, or it was getting, although it was indoors it seemed to be getting very dark and, I had ehm, and my blood pressure I don’t know, I don’t know what my blood pressure was but my pulse was, was going about a hundred and, at a hundred and seventy beats a minute and, I realised that was way too fast and I felt sort of dizzy so I went into emerge that time...

[Interviewer:] Alright. (.) So, did you feel like the Cloud DX tablet helped you at [that] incident?

[Respondent:] Yes, because like I didn’t, I was, I guess I was sort of dizzy I didn’t realize my pulse, when I saw my pulse was so high I knew I had to do something about it. Otherwise, I might not have gone in and I don’t what would’ve happened then.

Patient, remote monitoring

In summary, although the value propositions of the tool were not immediately clear to all stakeholders, those who engaged with the tool directly did feel the tool was valuable for the management of COPD, at least in certain cases. However, the primary value that appeared to be offered by the program arose not from the tool per se but from the relationship between patients and the respiratory therapist responsible for administering the program. We explore this point in the next section.

### Team: The Role of Relationships in the Reconfigured Service

The relationships between members of the health care team did not change in meaningful ways as a result of the implementation of the tool and the establishment of the remote-monitoring service. The respiratory therapist who administered the program was the primary point of contact for patients and would notify physicians, when appropriate, regarding issues arising for patients under their care. This process was not substantially different from how these providers communicated previously, as respiratory therapists had opportunities to interact with some patients before the beginning of this project in the context of a community-based exercise program and as recurring patients at the Centre for Respiratory Health and would communicate with physicians under similar circumstances as during the remote-monitoring project.

The relationships that were most important were those between the respiratory therapist administering the program and the patients enrolled in the program. Specifically, the relationships between the respiratory therapist and patients in the remote-monitoring program deepened and became an important component of the patients’ overall care. Many remote-monitoring patients spoke about the importance of this particular respiratory therapist for the value they experienced with the program, viewing the respiratory therapist not only as a source of information related to the tool itself, and COPD more generally, but as a person capable of supporting patients in solving health-related challenges of virtually any kind. The respiratory therapist corroborated this view by indicating that she had a good relationship especially with patients in the remote-monitoring group of the project:

So a lot of the patients call me because they know I’ll answer and it’s just to help get a prescription filled or sort of some other issue that they’re having... cause they know I’ll answer and have access to their physicians. They’ll call me over calling [the doctor’s] receptionist... Which maybe isn’t good, but, is also kind of good for [patients] because they feel more comfortable. Cause the relationship is there.

Allied Health Professional, very high interaction

Patients also reported the importance of being able to make contact with the respiratory therapist when questions arose regarding their health and health care. When asked what the most useful feature of the technology was, one patient stated:

Well, the point that you can, ehm, call somebody whenever you have a question, like [the respiratory therapist] you know. It’s very helpful because you have somebody to talk to about whatever the problem is. Whether it’s your breathing or your heart or what.

Patient, remote monitoring

These examples illustrate a fundamental mechanism by which the newly established service exhibited value for patients in the remote-monitoring group: through the relationship with the respiratory therapist administering the program. In this sense, although the tool was able to support the development of confidence and elicit appropriate responses to changing symptoms for patients across both self-monitoring and remote-monitoring groups, the value of the program extended beyond supporting the ability of patients to manage their COPD independently for the remote-monitoring group; it evolved to include a hidden care coordination function that was being performed by the respiratory therapist.

This care coordination function was not intended to be a part of the program from the outset and could be viewed as being a function of technological scope creep as a result of this respiratory therapist’s strong relationships with patients. Specifically, we use *technological scope creep* to refer to the unanticipated expanding scope of the respiratory therapists' work activities in relation to the function of the tool. The positive relationship between the respiratory therapist and remote-monitoring patients perhaps boosted their engagement with the tool but also fundamentally changed the nature of the remote-monitoring program. It was no longer simply a matter of monitoring vital signs and symptoms related to COPD, but at least for some patients, became a strategy to coordinate and access health services well beyond the original boundaries of the program. This technological scope creep is one component of the ways in which routines evolved as the program was implemented, which is addressed in the final section of the findings.

### Routine: Routines of Care and Hidden Work

The demand for changes in routines of care was very different for the different people involved in the project. For physicians, they had to change virtually nothing, and independently chose whether or not to act upon any information provided by the respiratory therapist administering the project. This meant that some physicians would act directly on the information provided and reach out to patients to follow up, whereas others would simply note the information but proceed with their usual approach to care.

Patients underwent a learning process as they came to interact with the new tool that became a part of their home environment during the project period. Although patients suggested that the learning process was a challenge in some cases, ultimately, the everyday use of the tool was viewed as largely unproblematic. One patient explained:

... I would make sure to get up a few minutes earlier than the time I needed to be up and kind of on my way out, to make sure I do have the time to do it. It’s not a big deal.

Patient, self-monitoring

In contrast to the relative ease experienced by physicians and patients, the respiratory therapists directly involved in the new program experienced drastic changes to their routines. In a sense, these changes were obvious and expected, as both respiratory therapists appointed to the program were fully aware that the program was just being established and represented net new work. However, the nature of the work they were ultimately required to carry out was not clear to them in advance. Two examples illustrate this point: extra documentation requirements and the growing communication demands arising from patient input through the tool.

In relation to documentation, the respiratory therapists were initially required to chart patient notes in the tool’s record system related to any care-related activity that took place through the tool. However, it quickly became apparent that this meant double charting and would not be sustainable were the program to become a part of usual care. One respiratory therapist described this as follows:

What we did was we actually charted in some of the patients’ notes on the Cloud DX project, sort of things that we thought were relevant. But then we found out we were actually double charting. Ehm, because we would have to chart in, you know, the EMR system. And so, I think that, you know, if it could be integrated into the system, like at the hospital, ehm that would be awesome. But at, at present, during the study, we, you know, we had to sort of do extra work just cause it wasn’t integrated.

Allied health professional, very high interaction

In this case, the known challenge of the lack of interoperability led to excessive workload on the part of the health care providers administering the program.

In relation to the communication demands arising from the program, these extended beyond the issue of patients calling the respiratory therapist for support in coordinating care more generally. The respiratory therapist also received calls related to technological challenges that were supposed to be directed to the vendor. Furthermore, the respiratory therapist would receive notifications indicating an exacerbation based on data that had been incorrectly entered and would need to call patients to follow up on those notifications only to discover that something had gone wrong in the data collection process (eg, they had responded to a survey incorrectly or the blood pressure cuff was not tightened properly). The respiratory therapist administering the program stated:

I can tell you that, ehm, in the remote monitoring group I had 116 incoming calls in 6 months and the self-monitoring group I had like 134 incoming calls in six months. So the total is, what, 240? So the remote-monitoring called me a little less than the self-monitoring. Ehm, and then I further broke that down into the reason for them calling and I’d say it’s like 77 were technological in the remote-monitoring and like 80 or so were technological [in self-monitoring]. And then the next highest one was just general health inquiries. Yeah, so, it’s, it’s mostly tech issues that they’re calling about. And they are told to call [the vendor], they just feel more comfortable calling me. And I generally just push it over to [the vendor]. In addition to those incoming calls, I had the notifications to respond to. So [sighs] I think in the three months for the remote monitoring group I had something, like, close to 300 notifications in six months, to respond to.

Allied health professional, very high interaction

The additional workload represented by these new routines was also not anticipated by managers. A manager responsible for considering the workload implication and sustainability of the program in the longer term commented:

[An important question is] if it’s something that’s sustainable and how do we integrate it into the day to day clinic activities? This study did not address that. So that will be something we’ll have to figure out.

Senior manager, little interaction

These instances of additional workload represent the establishment of new routines of care that were not anticipated at the outset of the program. These new routines quickly filled the time of the respiratory therapists responsible for the program, and added demands that would not be sustainable if the resources for staffing were held constant in the long-term. This finding represents the hidden work of technology implementation and remote-monitoring tools, which is not visible in advance of a program and might not be visible at all unless explicitly examined during the evaluation process.

## Discussion

### Principal Findings

Our findings direct attention toward two primary insights. Each insight helps to address the two primary objectives of our paper, which were to advance the Tool+Team+Routine heuristic for the adoption of digital health technologies and to clarify mechanisms by which remote monitoring may exert its value for patients. The first insight is the significance of interpersonal relationships for the functioning of this particular digital health technology in the context of delivering a comprehensive service of remote monitoring. The second is the expansion of routines of care and what we refer to as technological scope creep—the growing demands of health care providers’ everyday work as the technology requires new actions to fulfil newly generated demands.

### The Role of Relationships in Remote-Monitoring Initiatives

In our study, we found that one of the features of the service that most drove the successful engagement of patients with the technology was the relationship between the respiratory therapist administering the program and the patients who were enrolled. This finding resonates strongly with previous research [10,25] on the mechanisms by which digital health services have their effects. Vassilev et al [26] completed a realist review of the literature examining the specific causal mechanisms influencing the effectiveness of telehealth programs and found relationships to be one of three crucial mechanisms. They explained that new technologies introduce “new sources of relationality [26],” and thereby, “restructure existing relationships [26].”

In support of previous literature [7], our findings outline the value of such interpersonal relationships between the provider administering the program and the patients using the technology for the successful adoption of the service overall. In relation to the Tool+Team+Routine framework, this is an important point to emphasize; it is not just that such relationships enhance the adoption of the new technology, but that the relationships drive the development of an actual service arising from the technology itself. The technology is one component of a new program but relies on many other interacting components to become a part of a successfully delivered service (including people, technologies, and newly established processes).

In the context of service design, the adoption of new technologies stands to influence interpersonal relationship in at least two distinct ways. First, the technology might influence relationships among health care providers as they are required to collaborate in different ways than they previously had. We did not find this to be the case in our study, likely because the expectations that different health care providers had for one another did not change throughout the study period. However, this change to relationships among health care providers remains a possibility [25]. Second, the mechanisms by which the technology has its effects for patients are themselves determined in part by the quality of the relationships established between patients and the provider administering the program, and in this case, mediated by the technology. Emphasizing these relationships represents an important conceptual development for the Tool+Team+Routine heuristic, placing relationships in a more prominent position when taking a service design approach to promoting the adoption of technology-enabled service innovations. This point also reinforces the finding in the literature that the interpersonal relationship between the patient and the health care providers is a primary consideration in the effort to understand the ways in which remote monitoring has its effects [25].

### Technological Scope Creep

The second important finding emerging as an important direction for future research in our study is the phenomenon of technological scope creep. This point relates specifically to the *routine* element of the Tool+Team+Routine heuristic in a service design context, as it refers to changes in the routines of providers who must interact with the technology in ways that were not predicted at the outset. Furthermore, these newly added routines, in our study, were not accounted for in the time allocations assigned to providers responsible for administering the program via the technology, even though they might add additional value for patients. This point raises an important issue for the Tool+Team+Routine heuristic: in the effort to promote adoption of technologies for new services or service re-design, explicit effort should be made to anticipate technological scope creep and the demand for new routine practices that were not expected of the technology at the outset.

This point resonates with past work outlining how routine work practices change when new technologies are adopted [16,27,28]. However, past work has primarily emphasized the point that technologies restructure service arrangements [29]. We acknowledged this point in our past work [17] but extend this point now to acknowledge that even where work restructuring is explicitly anticipated in technology adoption projects, new technologies have a further potential to produce new work that is hidden from the view of management and evaluation as a result of not being considered as falling within the scope of the service itself. The technology evaluated in this study was intended to enable remote and self-monitoring of patients related specifically to COPD; however, patients ended up using the service as a general care coordination service. As a result of the relationships with the primary program administrator (the respiratory therapist), the scope of the service expanded to include this more general care coordination function. In effect, as a result of technological scope creep, the service itself expanded from its original function to take on a broader set of functions.

### Strengths and Limitations

This paper has applied a previously reported framework [17] to understand the implementation of a remote-monitoring technology into a health care environment. Although the patient participants recruited for this study might not reflect the needs and opinions of the entire COPD population, the sample of patients included provided in-depth insight into the value of the tool in the context of the service.

### Conclusions

The observations arising from our findings advance the Tool+Team+Routine heuristic for use in service design activities that involve technology adoption in health care services. Deep thinking about how the specific individuals administering the program will work to build relationships and how those relationships and other sources of activity might lead to technological scope creep present two often unanticipated challenges when implementing digital technologies in health care. Future work can reconcile these important influences on technology adoption and will examine strategies to account for them in advance of initiating technology implementation projects using a service design approach.

## Abbreviations

COPD: chronic obstructive pulmonary disease.

## References

[1] Global digital health market by major segment 2015-2020 | Statistic Internet. Statista.

[2] Buntin MB, Burke MF, Hoaglin MC, Blumenthal D (2011). The benefits of health information technology: a review of the recent literature shows predominantly positive results. Health Aff (Millwood).

[3] WHO (2011). Compendium of new and emerging health technologies. 1st ed.

[4] Huckvale K, Wang CJ, Majeed A, Car J (2019). Digital health at fifteen: more human (more needed). BMC Med.

[5] Fairbrother P, Pinnock H, Hanley J, McCloughan L, Sheikh A, Pagliari C, McKinstry B, TELESCOT programme team (2012). Continuity, but at what cost? The impact of telemonitoring COPD on continuities of care: a qualitative study. Prim Care Respir J.

[6] Kenealy TW, Parsons MJG, Rouse APB, Doughty RN, Sheridan NF, Hindmarsh JKH, Masson SC, Rea HH (2015). Telecare for diabetes, CHF or COPD: effect on quality of life, hospital use and costs. A randomised controlled trial and qualitative evaluation. PLoS One.

[7] Nissen L, Lindhardt T (2017). A qualitative study of COPD-patients' experience of a telemedicine intervention. Int J Med Inform.

[8] Vatnøy Torunn K, Thygesen E, Dale B (2017). Telemedicine to support coping resources in home-living patients diagnosed with chronic obstructive pulmonary disease: Patients' experiences. J Telemed Telecare.

[9] Greenhalgh T, Procter R, Wherton J, Sugarhood P, Hinder S, Rouncefield M (2015). What is quality in assisted living technology? The ARCHIE framework for effective telehealth and telecare services. BMC Med.

[10] Greenhalgh T, Wherton J, Papoutsi C, Lynch J, Hughes G, A'Court Christine, Hinder S, Procter R, Shaw S (2018). Analysing the role of complexity in explaining the fortunes of technology programmes: empirical application of the NASSS framework. BMC Med.

[11] Avgar A, Litwin A, Pronovost P (2012). Drivers and barriers in health IT adoption: a proposed framework. Appl Clin Inform.

[12] Ross J, Stevenson F, Lau R, Murray E (2016). Factors that influence the implementation of e-health: a systematic review of systematic reviews (an update). Implement Sci.

[13] Acharya Subrata, Werts Niya (2019). Toward the design of an engagement tool for effective electronic health record adoption. Perspect Health Inf Manag.

[14] Peek STM, Wouters EJ, Luijkx KG, Vrijhoef HJ (2016). What it Takes to Successfully Implement Technology for Aging in Place: Focus Groups With Stakeholders. J Med Internet Res.

[15] Keith R, Crosson J, O'Malley Ann S, Cromp D, Taylor E (2017). Using the Consolidated Framework for Implementation Research (CFIR) to produce actionable findings: a rapid-cycle evaluation approach to improving implementation. Implement Sci.

[16] May C (2013). Towards a general theory of implementation. Implement Sci.

[17] Shaw James, Agarwal Payal, Desveaux Laura, Palma Daniel Cornejo, Stamenova Vess, Jamieson Trevor, Yang Rebecca, Bhatia R Sacha, Bhattacharyya Onil (2018). Beyond "implementation": digital health innovation and service design. NPJ Digit Med.

[18] Stamenova Vess, Liang Kyle, Yang Rebecca, Engel Katrina, van Lieshout Florence, Lalingo Elizabeth, Cheung Angelica, Erwood Adam, Radina Maria, Greenwald Allen, Agarwal Payal, Sidhu Aman, Bhatia R Sacha, Shaw James, Shafai Roshan, Bhattacharyya Onil (2020). Technology-Enabled Self-Management of Chronic Obstructive Pulmonary Disease With or Without Asynchronous Remote Monitoring: Randomized Controlled Trial. J Med Internet Res.

[19] Connected Health Kit with Telemedicine Internet. Cloud DX.

[20] (2016). GOLD COPD Stages Internet. Lung Institute.

[21] Osterwalder A, Pigneur Y, Bernarda G, Smith A (2014). Value Proposition Design: How to Create Products and Services Customers Want.

[22] Bodenheimer T, Sinsky C (2014). From triple to quadruple aim: care of the patient requires care of the provider. Ann Fam Med.

[23] Bradley EH, Curry LA, Devers KJ (2007). Qualitative data analysis for health services research: developing taxonomy, themes, and theory. Health Serv Res.

[24] Braun V, Clarke V, Cooper H, Camic PM, Long DL, Panter AT, Rindskopf D, Sher KJ (2012). Thematic analysis. APA Handbook of Research Methods in Psychology, Vol 2: Research Designs: Quantitative, Qualitative, Neuropsychological, and Biological.

[25] Shaw J, Shaw S, Wherton J, Hughes G, Greenhalgh T (2017). Studying Scale-Up and Spread as Social Practice: Theoretical Introduction and Empirical Case Study. J Med Internet Res.

[26] Vassilev I, Rowsell A, Pope C, Kennedy A, O?Cathain A (2015). Salisbury C Assessing the implementability of telehealth interventions for self-management support: a realist review. Implementation Science.

[27] Nicolini D (2006). The work to make telemedicine work: a social and articulative view. Soc Sci Med.

[28] Nicolini D (2016). Stretching out and expanding work practices in time and space: The case of telemedicine. Human Relations.

[29] Cresswell K, Worth A, Sheikh A (2010). Actor-Network Theory and its role in understanding the implementation of information technology developments in healthcare. BMC Med Inform Decis Mak.

